# Body image, self-esteem, emotion regulation, and eating disorders in adults: a systematic review

**DOI:** 10.1007/s40211-025-00544-4

**Published:** 2025-08-20

**Authors:** Marzieh Abdoli, Elisabeth Schiechtl, Marco Scotto Rosato, Barbara Mangweth-Matzek, Paolo Cotrufo, Katharina Hüfner

**Affiliations:** 1 Observatory on Eating Disorders, Department of Psychology, University of Campania “Luigi Vanvitelli”, 31, 81100 Caserta, Italy; 2 University Clinic for Psychiatry II, Department of Psychiatry, Psychotherapy, Psychosomatics and Medical Psychology, Medical University of Innsbruck, Innsbruck, Austria

**Keywords:** Eating disorders, Body image, Self-esteem, Emotion regulation, Adults, Essstörungen, Körperbild, Selbstwert, Emotionsregulation, Erwachsene

## Abstract

This systematic review examines the connections between eating disorders, body image disturbance, self-esteem, and emotion regulation in adults. Following the PRISMA guidelines, a systematic search of PubMed, Scopus, and Web of Science databases was conducted for articles published between 2010 and June 2024. Studies were included if they involved participants aged 18 years and older, employed validated tools for measuring the variables, and presented original research that specifically addressed these psychological factors. Out of 1117 records, six studies met the inclusion criteria, with mostly female samples and a focus on body image, self-esteem, and emotion regulation in relation to eating disorders. The results indicate that body dissatisfaction is closely related to disordered eating behaviors, with a significant link to lower self-esteem and difficulties in emotion regulation. Obese individuals with binge eating disorder (BED) were found to have more negative attitudes toward obesity and greater levels of depression than their non-BED counterparts. Women with bulimia nervosa showed higher emotion-focused coping, which is associated with low self-worth. Differences in gender were evident, with women exhibiting greater vulnerability to body image dissatisfaction and emotion dysregulation. The results show that treatment for adults with eating disorders should focus on the enhancement of self-esteem, the improvement of body image perception, and the development of adaptive emotion regulation strategies. Lastly, practicing self-compassion techniques in psychotherapy could improve the treatment process for patients suffering from eating disorders, low self-esteem, emotion dysregulation, and body image disturbance. Future studies should investigate these variables in various non-Western cultural contexts for better understanding and clinical intervention for the adult population.

## Abstract (de)

In der vorliegenden systematischen Übersichtsarbeit werden die Verbindungen zwischen Essstörungen, Störungen des Körperbildes, Selbstwert und Emotionsregulation bei Erwachsenen dargelegt. Nach den PRISMA-Leitlinien (Preferred Reporting Items for Systematic Reviews and Meta-Analyses) wurde eine systematische Suche nach zwischen 2010 und Juni 2024 publizierten Artikeln in den Datenbanken PubMed, Scopus und Web of Science durchgeführt. Studien wurden dann einbezogen, wenn die Teilnehmer:innen mindestens 18 Jahre alt waren, validierte Instrumente zur Messung der Variablen eingesetzt und Original-Forschungsergebnisse dieser psychologischen Faktoren präsentiert wurden. Von 1117 identifizierten Studienerfüllten sechs die Einschlusskriterien. Meist handelte es sich um weibliche Stichproben und einen Fokus auf Körperbild, Selbstwert und emotionaler Regulation im Zusammenhang mit Essstörungen. Die Ergebnisse zeigen, dass Unzufriedenheit mit dem Körper in enger Beziehung zu gestörtem Essverhalten steht, dabei besteht ein signifikanter Zusammenhang mit niedrigerem Selbstwert und Schwierigkeiten bei der emotionalen Regulation. Bei adipösen Personen mit Binge Eating Störung („binge eating disorder“, BED) fanden sich mehr negative Haltungen zu Adipositas und ein höheres Niveau an Depression im Vergleich zu entsprechenden Personen ohne BED. Frauen mit Bulimia nervosa zeigten höhere emotionsfokussierte Bewältigungsstrategien, was mit niedrigem Selbstwert einhergeht. Unterschiede beim Geschlecht waren evident, Frauen wiesen eine größere Vulnerabilität für Unzufriedenheit mit dem Körperbild und emotionale Dysregulation auf. Die Ergebnisse zeigen, dass die Therapie für Erwachsene mit Essstörungen den Fokus auf die Erhöhung des Selbstwerts, Verbesserung der Körperbildwahrnehmung und Entwicklung von adaptiven emotionalen Regulationsstrategien legen sollte. Schließlich kann das Praktizieren von Selbstmitgefühlstechniken in der Psychotherapie den Behandlungsprozess für Patienten mit Essstörungen, niedrigem Selbstwert, emotionaler Dysregulation und Störung des Körperbildes verbessern. In zukünftigen Studien sollten diese Variablen in verschiedenen nichtwestlichen kulturellen Kontexten mit dem Ziel eines besseren Verständnisses und besserer klinischer Interventionen für die erwachsene Bevölkerung untersucht werden.

## Introduction

Eating disorders are complicated and varied psychological illnesses with potentially disastrous effects on individuals’ mental and physical health [59]. Profound disturbances in eating habits and a strong preoccupation with body weight and shape can lead to disorders such as anorexia nervosa, bulimia nervosa, and binge eating disorder, causing significant disease and mortality [21, 33]. Individuals suffering from anorexia nervosa deliberately starve themselves and have a strong fear of gaining weight, which can be intensely harmful [43, 48]. This leads to severe malnutrition and failure of many organs, which may be life-threatening [42, 70]. Bulimia nervosa is characterized by repeated periods of excessive eating, followed by actions such as vomiting or excessive activity to compensate for binge eating [20, 26, 60]. These behaviors may result in medical complications [20, 70]. Binge eating disorder, the most widespread eating disorder, is characterized by repeated periods of excessive eating without subsequent compensatory actions, often leading to obesity and its relative comorbidities, such as type 2 diabetes and cardiovascular disease [2, 63]. The connection between eating disorders and body image is well established, with a poor body image often acting as both a precursor and a perpetrator of disordered eating habits [4, 30]. Body image encompasses an individual’s views, thoughts, and emotions about their physical appearance. Negative body image is discontent with one’s body size, shape, or weight [23, 64]. Feelings of discontentment may result in detrimental actions focused on modifying physical appearance, which in turn can provoke or intensify symptoms of eating disorders [62, 68]. Furthermore, the negative psychological consequences of having a poor body image go beyond just having an eating disorder. A poor body image is closely linked to experiencing emotional discomfort, such as sadness, anxiety, and a lack of self-worth [35, 50, 54]. Emotions and their management are crucial in the formation and maintenance of eating disorders. Individuals with disordered eating habits often have difficulty controlling their emotions and resort to utilizing food-related actions as a means of dealing with unpleasant feelings like stress, depression, or anger [14, 53]. Emotional dysregulation has a role in both the development and continuation of eating disorders by strengthening unhealthy ways of dealing with emotions [40, 46]. Low self-esteem is another key factor intertwined with body image and emotion regulation in the context of eating disorders. It influences and is influenced by the other variables, creating a feedback loop that can exacerbate disordered eating behaviors [13]. Individuals with low self-esteem are more vulnerable to the effects of negative body image and emotional distress, which can lead to the adoption of harmful eating behaviors as a means of managing these feelings [15, 24]. This complex interplay highlights the need for comprehensive interventions that address all these factors to treat and prevent eating disorders effectively [38, 52, 74]. While extensive research has examined the relationship between eating disorders and these psychological variables in adolescents [27, 39, 56, 57, 61], less attention has been paid to adults. Existing studies often focus on individual factors, such as the connection between eating disorders and body image [67] or body image and self-esteem [16, 34], without considering the interconnectedness of all these variables within adult populations.

## Method

### Study protocol

**Fig. 1:**
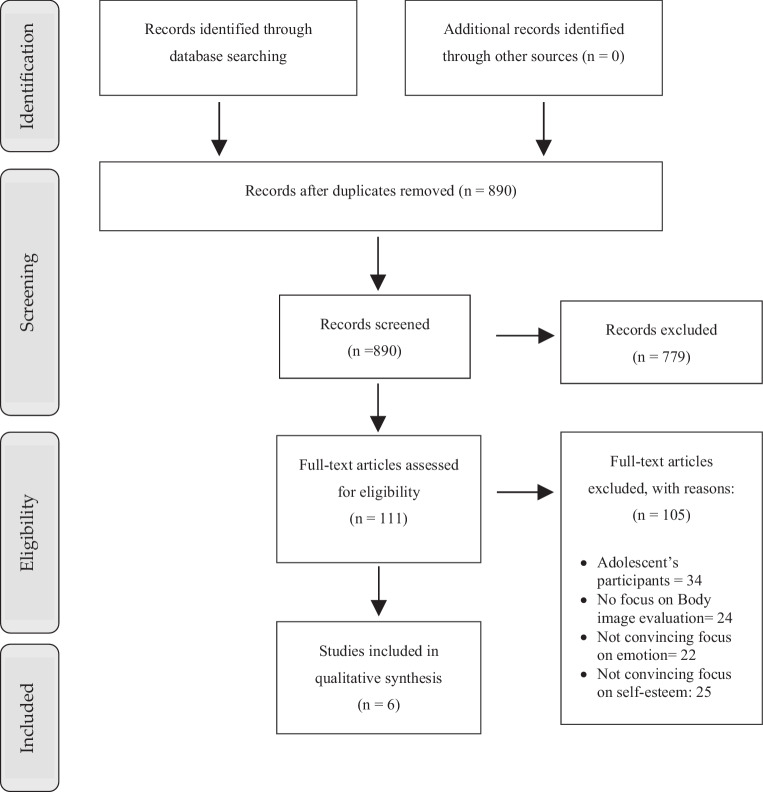
PRISMA flow diagram

This systematic review followed a structured approach based on the Preferred Reporting Items for Systematic Reviews and Meta-Analyses (PRISMA) guidelines (Fig. 1), focusing on exploring the relationships between eating disorders, body image, emotion regulation, and self-esteem in adults [49]. The study protocol was designed to identify, evaluate, and synthesize research that examined the connections between these psychological factors in adult populations. The review specifically targeted studies involving individuals aged 18 years and older, excluding research focused on adolescents. A systematic search was conducted across three databases: PubMed, Scopus, and Web of Science. A standardized set of search terms was used across all three databases. Keywords related to “body image,” “emotional regulation,” “eating disorders,” and “self-esteem” were used to capture relevant studies published between 2010 and June 2024 (Table 1). The same core terms and time limits were applied to ensure methodological consistency across the databases. The protocol of this review is registered in the PROSPERO database (CRD42024562669).

**Table 1:** Database, record, and index

Database	Search terms	Articles identified
PubMed	((body image OR body perception OR body attitude OR body dissatisfaction OR body concerns OR body image disturbance OR negative body image OR body image issues OR body discontent OR negative body attitudes OR body image dissatisfaction) AND (emotion regulation OR emotional control OR emotional management OR affect regulation OR mood regulation OR emotional adjustment OR emotion processing OR emotional reactivity OR emotional resilience OR emotional response OR regulating emotions OR affective control OR managing emotions OR emotional balance OR affect dysregulation OR affect recognition OR alexithymia OR emotional awareness OR emotional dysregulation OR emotional expression* OR emotional modulation) AND (eating disorders OR anorexia OR bulimia OR binge eating OR eating pathology OR disordered eating OR compulsive eating OR eating habits OR food behavior OR feeding disorders OR anorexia nervosa OR bulimia nervosa OR binge eating disorder OR dietary restraint OR food intake disorders OR external eating OR affective eating OR eating attitude OR eating behavior OR eating habit OR eating practice OR eating style) AND (self-esteem OR self-worth OR self-respect OR self-perception OR self-confidence OR self-efficacy OR self-value OR self-evaluation)) AND (adults OR adult population OR mature individuals OR adulthood OR mature population OR over 18 years OR over 18 OR 18 years older OR adult age group OR aged 18+ OR legal age) NOT (Review[Publication Type])	393
Scopus	TITLE-ABS-KEY(“body image” OR “body perception” OR “body attitude” OR “body dissatisfaction” OR “body concerns” OR “body image disturbance” OR “negative body image” OR “body image issues” OR “body discontent” OR “negative body attitudes” OR “body image dissatisfaction”) AND (“emotion regulation” OR “emotional control” OR “emotional management” OR “affect regulation” OR “mood regulation” OR “emotional adjustment” OR “emotion processing” OR “emotional reactivity” OR “emotional resilience” OR “emotional response” OR “regulating emotions” OR “affective control” OR “managing emotions” OR “emotional balance” OR “affect dysregulation” OR “affect recognition” OR “alexithymia” OR “emotional awareness” OR “emotional dysregulation” OR “emotional expression*” OR “emotional modulation”) AND (“eating disorders” OR “anorexia” OR “bulimia” OR “binge eating” OR “eating pathology” OR “disordered eating” OR “compulsive eating” OR “eating habits” OR “food behavior” OR “feeding disorders” OR “anorexia nervosa” OR “bulimia nervosa” OR “binge eating disorder” OR “dietary restraint” OR “food intake disorders” OR “external eating” OR “eating attitude” OR “eating behavior” OR “eating habit” OR “eating practice” OR “eating style”) AND (“self-esteem” OR “self-respect” OR “self-confidence”) AND (“adults” OR “adult population” OR “mature individuals” OR “adulthood” OR “mature population” OR “over 18 years” OR “over 18” OR “adult age group” OR “aged 18+” OR “legal age”) AND NOT DOCTYPE (“re”) AND PUBYEAR > 2009 AND PUBYEAR < 2025 AND (LIMIT-TO (SUBJAREA,“PSYC”) OR LIMIT-TO (SUBJAREA,“SOCI”) OR LIMIT-TO (SUBJAREA,“ARTS”) OR LIMIT-TO (SUBJAREA,“HEAL”) OR LIMIT-TO (SUBJAREA,“MULT”)) AND (LIMIT-TO (LANGUAGE,“English”)) AND (LIMIT-TO (DOCTYPE,“ar”)) AND (LIMIT-TO (SRCTYPE,“j”)) AND (LIMIT-TO (PUBSTAGE,“final”)	555
Web of Science	(ALL = (“body image*” OR “body dissatisf*” OR “body perception*” OR “body attitude*” OR “body concern*” OR “body image disturbance*” OR “negative body image*” OR “body image issue*” OR “body discontent*” OR “negative body attitude*” OR “body image dissatisfaction*”) AND ALL = (“emotion*” OR “emotional control” OR “emotional management” OR “emotional adjustment” OR “emotion processing” OR “emotional reactivity” OR “emotional resilience” OR “emotional response” OR “regulating emotion*” OR “affective control” OR “managing emotion*” OR “emotional balance” OR “affect dysregulation” OR “affect recognition” OR “alexithymia” OR “emotional awareness” OR “emotional dysregulation” OR “emotional expression*” OR “emotional modulation”) AND ALL = (“eating disorder*” OR “eating behav*” OR “anorexia*” OR “bulimia*” OR “binge eating” OR “eating pathology” OR “disordered eating” OR “compulsive eating” OR “food behavior*” OR “feeding disorder*” OR “anorexia nervosa” OR “bulimia nervosa” OR “binge eating disorder” OR “dietary restraint” OR “food intake disorder*” OR “external eating” OR “eating attitude*” OR “eating practice*” OR “eating style*”) AND ALL = (“self-” OR “self esteem” OR “self respect” OR “self confidence” OR “self perception” OR “self evaluation” OR “self worth” OR “self efficacy”) AND ALL = (“adult” OR “mature individual*” OR “older adult*” OR “over 18 years” OR “aged 18+” OR “legal age” OR “elderly” OR “senior” OR “middle-aged” OR “older population”)) NOT (DT = Review)	169

### Eligibility criteria

The eligibility criteria for this systematic review were established to ensure the inclusion of studies that specifically addressed the relationships between eating disorders, body image, emotion regulation, and self-esteem in adult populations. The criteria applied included:

- Studies must involve adult participants aged 18 years and older. Research focusing exclusively on adolescents or children was excluded to maintain a clear focus on adult populations.
- Included studies must investigate the relation between eating disorders (e.g., anorexia nervosa, bulimia nervosa, binge eating disorder, obesity), body image disturbances, and at least one of the following psychological factors: emotion regulation or self-esteem with consideration of emotions, or self-esteem. Studies involving both clinical populations (those diagnosed with eating disorders) and nonclinical populations (those exhibiting dysfunctional eating habits without a formal diagnosis) were included.
- Only original research articles, including quantitative, qualitative, and mixed methods studies, were considered.
- Studies published between 2010 and June 2024 were eligible for inclusion.
- Only studies published in English were included to ensure clarity and consistency in data interpretation. Exclusion criteria were also applied to refine the selection: (i) non-adult populations; (ii) studies that did not include eating disorders and did not provide a clear and specific focus on body image, together with either emotionregulation or self-esteem.

A “clear and specific focus” was operationalized as the explicit assessment or measurement of body image disturbance in combination with validated scales measuring either emotional regulation (such as emotion regulation questionnaires, alexithymia scales, emotional awareness scales) or self-esteem (such as the Rosenberg Self-Esteem Scale, self-worth or self-confidence measures). Studies that mentioned these constructs superficially, without a dedicated methodology or validated assessments, were considered insufficiently focused and therefore excluded.

### Study selection

After the initial search, records from the database searches were imported into reference management software, where duplicates were removed using the reference management software Zotero (v. 6.0.26), (Corporation for Digital Scholarship, Vienna, VA, USA). This resulted in a total of 890 articles. The screening process was conducted in two stages by two independent reviewers (M.A. and M.S.R). The titles and abstracts of the 890 records were reviewed to assess their relevance to the objectives of this review. Studies were excluded at this stage if they did not meet the inclusion criteria. This stage resulted in the exclusion of 779 articles. The remaining 111 articles were subjected to a full-text review. In this phase, articles were thoroughly evaluated according to the predefined eligibility criteria. Two researchers independently reviewed all studies and discussed the inclusion and exclusion of each study. A third reviewer acted as a referee in cases of disagreement. Inter-rater reliability (Cohen’s kappa) was not formally calculated due to resource constraints and the small number of included studies. However, disagreements were minimal, and consensus was reached through detailed discussion between reviewers and referee involvement. Individual work on syntax and bias prevention was carried out to ensure the integrity of the review process. Articles were excluded for reasons such as focusing on adolescent populations (34 articles), not adequately addressing body image evaluation (24 articles), insufficient focus on emotional factors (22 articles), or lacking convincing focus on self-esteem (25 articles). This rigorous process resulted in the final inclusion of six studies.

### Synthesis and analysis of data

The data synthesis involved a narrative approach due to the heterogeneity of the included studies in terms of their methodologies, populations, and measured outcomes. The results were organized into thematic categories aligned with the study’s objectives: eating disorders, body image, emotion regulation, self-esteem, gender differences. A qualitative synthesis integrated findings across studies, focusing on identifying common patterns and themes. Where applicable, quantitative data were also summarized, particularly when comparing the prevalence and severity of eating disorders across different populations. The synthesis aimed to understand the interrelations between the core variables of interest.

## Result

### Study characteristics

The six studies included in this review varied in their aims, methodologies, and geographic locations, providing a broad perspective on the interconnections between eating disorders, body image, emotion regulation, and self-esteem. All studies were published between 2010 and June 2024 and focused on adult populations, with a mix of both clinical and community samples. The tools used in these studies were well-established in the field, including the Eating Disorder Examination (EDE), Rosenberg Self-Esteem Scale (RSES), and Beck Depression Inventory (BDI), among others (see Table 2). The results consistently highlighted significant associations between the core variables of interest, with variations observed across different Western geographic contexts, including the United States, Norway, Canada, and Germany.

**Table 2:** Study characteristics

Study	Authors	Year	Aim	Tools	Sample size	Population type	Statistical methods	Result	Country
An examination of weight bias among treatment-seeking obese patients with and without binge eating disorder	(Barnes et al., [5])	2014	To examine weight bias among obese patients with and without BED seeking treatment in primary care settings	Eating Disorder Examination (EDE), Attitudes Towards Obese People (ATOP) Scale, Beck Depression Inventory (BDI)	*N* = 221 (BED = 168; NBO = 53)	Clinical (treatment-seeking obese patients with/without BED)	Two-way ANOVA, *t* tests, bivariate correlations	Patients with BED reported greater negative attitudes toward obese people than those without BED. Weight bias was related to higher depression and eating disorder psychopathology for all patients	USA
Relationship between body dissatisfaction and disordered eating: mediating role of self-esteem and depression	(Brechan & Kvalem, [7])	2015	To examine the mediating roles of self-esteem and depression in the relationship between body dissatisfaction and disordered eating behaviors	Body-Image Ideals Questionnaire (BIQ), Rosenberg Self-Esteem Scale (RSES), Beck Depression Inventory-II (BDI-II), Eating Disorder Examination Questionnaire (EDE-Q)	*N* = 320 (192 females, 128 males)	Non-clinical (college students)	Structural equation modeling (SEM), bootstrapping, regression	Body dissatisfaction and body image importance impact self-esteem and depression, which in turn affect disordered eating behaviors. Gender differences were observed in the effects of depression	Norway
Body esteem as a common factor of a tendency toward binge eating and sexual dissatisfaction among women: the role of dissociation and stress response during sex	(Castellini et al., [10])	2017	To investigate the association between body esteem, binge eating, and sexual dissatisfaction, focusing on dissociation and stress response during sexual activity	Clinician-Administered Dissociative States Scale (CADSS), Sexual Satisfaction Scale-Women (SSS-W), Body Esteem Scale for Adolescents and Adults (BESAA), Eating Attitudes Test (EAT-26), Cortisol assessment via saliva samples	*N* = 60 (women; 33 BED group, 27 controls)	Clinical (BED patients and controls)	Pearson correlations, *t* tests, regression analysis	Women with higher tendencies toward binge eating and dissociation during sexual experiences showed higher levels of sexual distress and an altered cortisol response to sexual stimuli, indicating a stress response. Body esteem, particularly weight satisfaction, was significantly linked to dissociation	USA
Self-compassion moderates the relationship between body mass index and both eating disorder pathology and body image flexibility	(Castellini et al., [10])	2014	To examine whether self-compassion moderates the relationship between BMI and eating disorder pathology and body image flexibility	Self-Compassion Scale (SCS), Rosenberg Self-Esteem Inventory (RSE), Body Image-Acceptance and Action Questionnaire (BI-AAQ), Eating Disorder Examination Questionnaire 6.0 (EDE‑Q 6.0)	*N* = 154 (female undergraduates)	Nonclinical (university students)	Pearson correlations, hierarchical multiple regression	Self-compassion was found to moderate the relationship between BMI and eating disorder pathology, as well as body image flexibility. Higher self-compassion reduced the negative impact of BMI on these outcomes	Canada
Body image disturbance in binge eating disorder: a comparison of obese patients with and without binge eating disorder regarding the cognitive, behavioral, and perceptual component of body image	(Lewer et al., [37])	2017	To examine whether different components of body image disturbance manifest in BED similarly to other eating disorders, comparing obese females with and without BED	Eating Disorder Inventory‑2 (EDI-2), Eating Disorder Examination-Questionnaire (EDE-Q), Body Image Avoidance Questionnaire (BIAQ), Body Checking Questionnaire (BCQ), Beck Depression Inventory (BDI), Rosenberg Self-Esteem Scale (RSES), Symptom Checklist K‑9, Digital Photo Distortion Technique	*N* = 86 (BED = 41; non-BED = 45)	Clinical (obese women with and without BED)	MANOVA, ANOVA, Pearson correlations	Obese individuals with BED show greater body image disturbance than those without BED, with significant differences in cognitive-affective components and discrepancy between actual and ideal body dimensions	Germany
Examination of situational coping in bulimic women in a social interaction	(Wölfges et al., [71])	2011	To examine situational coping in bulimic women during a social interaction and to investigate possible moderating factors	Eating Disorder Examination (EDE) interview, Dysfunctional Attitude Scale (DAS), Toronto Alexithymia Scale (TAS), Rosenberg Self-Esteem Scale (RSS), Stress-Verarbeitungs-Fragebogen 42-aktuell (SVF 42-ak)	*N* = 53 (BN = 26, CG = 27)	Clinical (bulimia nervosa patients) vs. nonclinical controls	MANOVA, MANCOVA, regression analysis	Women with bulimia showed significantly higher emotion-oriented coping compared to controls, with self-esteem influencing coping style	Germany

*BED* binge eating disorder

### Sample characteristics

The participants’ mean ages ranged from early adulthood (20.2 years) to middle adulthood (49.66 years). The gender distribution was skewed toward females, with all studies either predominantly or exclusively including women. Ethnicity was reported in some studies, with diverse populations including White, African American, Hispanic, and Asian American participants. This allowed for an examination of cultural differences in the manifestation and perception of body image and eating disorders (see Table 3).

**Table 3:** Sample characteristics

Authors	Year	Age (years)	Gender	Ethnicity
(Barnes et al., [5])	2014	BED Group: M = 46.57 (SD = 10.61); NBO Group: M = 49.66 (SD = 9.95)	BED Group: 75% Women, 25% Men; NBO Group: 81% Women, 19% Men	BED Group: 45.2% White, 32.1% African American, 13.7% White Hispanic, 2.4% Asian American, 1.8% African American Hispanic, others; NBO Group: 66.0% White, 26.4% African American, 5.7% White Hispanic, others
(Brechan & Kvalem, [7])	2015	Mean age: 24.12 (Women), 24.51 (Men)	65% Women, 35% Men	Not specified
(Castellini et al., [10])	2017	25–35 years (M = 29.8 ± 5.2)	100% Women	Not specified
(Kelly et al., [32])	2014	Mean age: 20.2 (SD = 3.49)	100% Women	48.3% Caucasian, 19.4% South Asian, 12.9% East Asian, 6.5% Southeast Asian, 3.2% Black/African, 3.2% Bi-racial, 2.6% West Indian/Caribbean, 1.3% Hispanic, 1.3% Middle Eastern, 0.7% Aboriginal, 0.7% Other
(Lewer et al., [37])	2017	Mean age: 43.94 (BED group), 44.11 (non-BED group)	Female	Not specified
(Wölfges et al., [71])	2011	25.7 ± 6.1 (combined)	Female	Not specified

*BED* binge eating disorder, *NBO* no binge eating disorder

**Table 4:** Mixed Method Appraisal Tool (MMAT)

Authors	Screening questions		Category of study design									
			2. Quantitative randomized controlled trials					4. Quantitative descriptive				
	S1 Are there clear research questions?	S2 Do the collected data allow one to address the research questions?	2.1	2.2	2.3	2.4	2.5	4.1	4.2	4.3	4.4	4.5
(Barnes et al., [5])	Y	Y	–	–	–	–	–	Y	Y	Y	C	Y
(Brechan & Kvalem, [7])	Y	Y	–	–	–	–	–	Y	Y	Y	Y	Y
(Castellini et al., [10])	Y	Y	–	–	–	–	–	Y	C	Y	Y	Y
(Kelly et al., [32])	Y	Y	–	–	–	–	–	Y	Y	Y	C	Y
(Lewer et al., [37])	Y	Y	–	–	–	–	–	Y	C	Y	C	Y
(Wölfges et al., [71])	Y	Y	–	–	–	–	–	Y	Y	Y	Y	Y

### Assessments of risk of bias and quality

The risk of bias and quality of the included studies were assessed using the Mixed Methods Appraisal Tool (MMAT), a validated tool designed for appraising empirical studies across qualitative, quantitative, and mixed methods research [28]. The MMAT was chosen for its comprehensive approach, enabling standardized assessments across different study designs (Table 4). Common sources of bias included potential selection bias, particularly in studies with self-selected samples from clinical or community settings, and measurement bias in studies using self-report questionnaires prone to social desirability bias. Despite these concerns, the overall quality of the studies was considered high, with most scoring well across the MMAT criteria. Including validated measurement tools and rigorous statistical analyses helped mitigate some inherent biases. Each study was evaluated according to criteria relevant to its design category, with quantitative descriptive studies assessed for clarity in research questions, sampling strategy, sample representativeness, measurement tool adequacy, and statistical analysis suitability. Quantitative nonrandomized studies were evaluated for group comparability, control of confounding variables, and the appropriateness of the intervention or exposure. Two researchers independently reviewed all studies and discussed all the included and excluded studies.

### Eating disorders

**Fig. 2:**
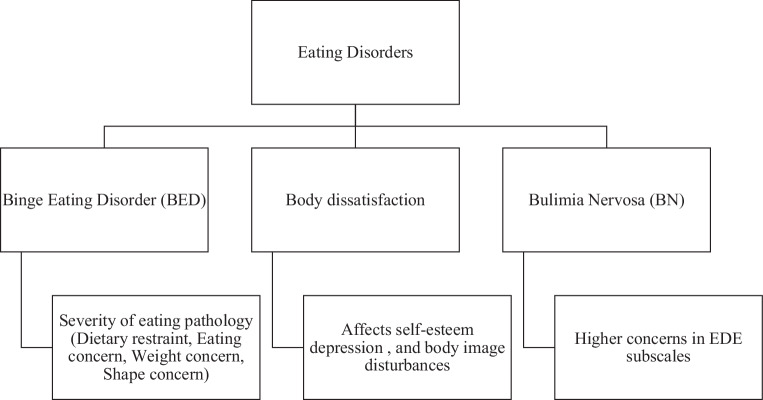
Interconnections in eating disorders. *BED* binge eating disorder, *BN* bulimia nervosa, *EDE* Eating Disorder Examination

This review examined six studies that investigated different eating disorders, with a particular emphasis on binge eating disorder (BED) and bulimia nervosa (BN). It was found that individuals with BED showed more severe eating disorder pathology compared to those without BED (Fig. 2). This was demonstrated by the increased scores on the subscales of the Eating Disorder Examination (EDE), which included dietary restraint, eating concern, weight concern, and shape concern [5]. Similarly, in another study, women diagnosed with BN displayed significantly higher levels of concern across all EDE subscales compared to controls, suggesting a more pronounced manifestation of disordered eating behaviors [71]. In addition, the study examined the connection between body dissatisfaction and disordered eating. The results revealed that body dissatisfaction had an indirect impact on disordered eating behaviors by affecting self-esteem and depression levels [7]. The studies collectively emphasize the connection between disordered eating behaviors, whether in BED or BN, and psychological factors. These disorders are influenced by factors such as low self-esteem, depression, and body image disturbance, which contribute to their severity [10, 32].

### Body image

**Fig. 3:**
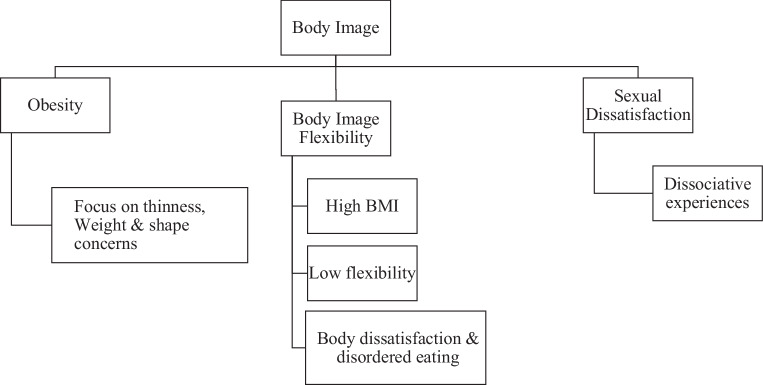
Body image and its associated factors. *BMI* body mass index

Body image disturbances were a consistent theme across all studies, especially in relation to their impact on eating disorders. Obese individuals with BED displayed a significant focus on thinness, along with heightened weight and shape concerns, indicating the presence of severe body image disturbances within this particular group [37]. The study examined the idea of body image flexibility, which refers to the capacity to embrace negative thoughts about one’s body. The results revealed (Fig. 3) a correlation between higher BMI and lower body image flexibility, leading to increased levels of body dissatisfaction and disordered eating behaviors [32]. In addition, it is worth noting that body esteem, specifically weight satisfaction, has been found to have a significant correlation with sexual dissatisfaction and dissociative experiences during sex. This highlights the important role that body image plays in both psychological and sexual well-being [10]. The studies collectively showed that body dissatisfaction and related concerns are not only common in individuals with eating disorders but also play a crucial role in driving these disorders. In addition, research consistently supports the link between body image and eating disorders. Body dissatisfaction often leads to behaviors such as restricted eating, compensatory actions, and binge eating. This connection is especially prevalent in individuals with higher BMI or those diagnosed with BED and BN [5, 71].

### Emotion

**Fig. 4:**
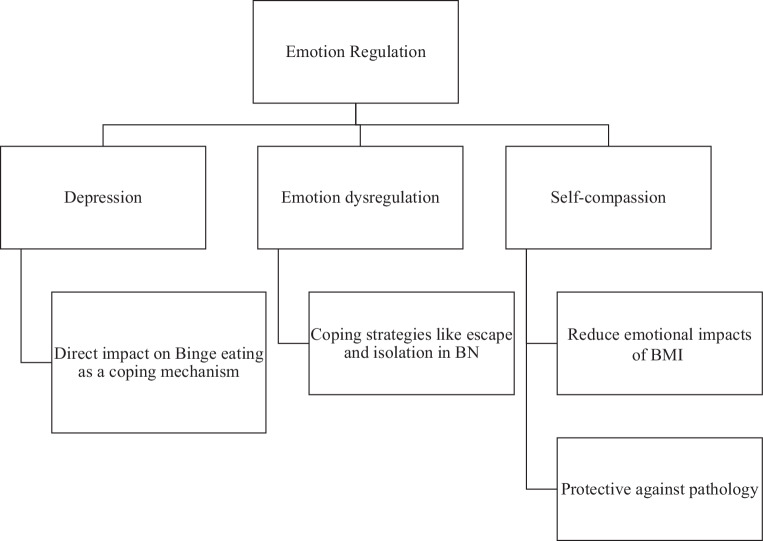
Role of emotion regulation in eating disorders

Emotionl regulation, or its lack thereof, plays a significant role in the development of eating disorders and body image issues (Fig. 4). Depression, a recurring theme, has been found to directly impact binge eating, as individuals turn to disordered eating behaviors as a way to cope with negative emotions [7]. Women with BN displayed emotional dysregulation through their use of coping strategies focused on emotions, such as seeking escape and isolating themselves socially. These behaviors were found to be significantly more prominent compared to the control group [71]. Self-compassion was found to play a crucial role in mitigating the negative emotional impacts of higher BMI, thus acting as a protective factor in the relationship between BMI and eating disorder pathology [32]. In addition, there was a connection between emotional stress, dissociative experiences, and binge eating in the context of sexual activities [10]. These studies emphasize the significance of emotion regulation in the development and persistence of eating disorders and body image concerns.

### Self-Esteem

**Fig. 5:**
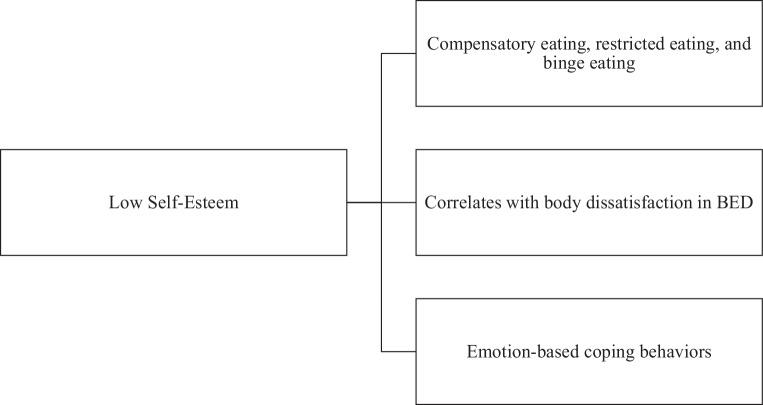
Role of self-esteem. *BED* binge eating disorder

Self-esteem played a crucial role in connecting body image, emotions, and eating disorders (Fig. 5). Research has shown a clear connection between low self-esteem and behaviors such as compensatory eating, restrained eating, and binge eating. This connection is especially prevalent among women [7]. In individuals with BED who are overweight, having low self-esteem was found to be linked to feeling more dissatisfied with their body image. This dissatisfaction can contribute to the continuation of disordered eating behavior [37]. The studies also found that self-compassion may be more important than self-esteem in preventing negative effects associated with body image and eating disorders. This suggests that it may be beneficial to focus on promoting self-compassion rather than traditional self-esteem interventions [32]. Furthermore, in the case of women with BN, having low self-esteem was found to be a predictor of relying on emotion-oriented coping behaviors. It is worth noting that these coping mechanisms are generally less effective in managing stress and can even worsen symptoms of eating disorders [71]. These findings highlight the importance of self-esteem and related constructs, such as self-compassion, in the psychological well-being of individuals with eating disorders. It suggests that enhancing these factors may result in more positive treatment outcomes.

### Gender differences

Studies have found that women may be more susceptible to the negative effects of body image dissatisfaction and emotional dysregulation on eating disorders. Research has shown that there are differences in how men and women experience body dissatisfaction, self-esteem, and symptoms of depression. These differences can contribute to the development of disordered eating behaviors, such as binge eating and restrained eating [7]. On the other hand, men seemed to adopt various coping strategies when faced with depressive symptoms, such as turning to alcohol instead of restricting their eating habits [5]. The studies also emphasized the importance of self-compassion in influencing the connection between BMI and eating disorder pathology in women. This suggests that interventions aimed at boosting self-compassion may have a greater impact on women’s response to treatment [32].

### Secondary outcomes

The six included studies also offered a further understanding of the relationship between psychological factors and eating disorders. As an example, the study found that weight bias plays a significant role in causing psychological distress and disordered eating behaviors, especially in individuals with BED [5]. Another secondary outcome examined was the role of dissociation during sexual activities. The findings indicate that women who use dissociation as a coping mechanism may be more prone to binge eating and sexual dissatisfaction [10]. The studies also emphasized the significance of considering cultural and racial differences in the treatment of eating disorders. African American participants reported lower levels of weight bias compared to their White counterparts, suggesting that cultural factors may play a role in the internalization of body image concerns [37]. These secondary outcomes indicate that a comprehensive treatment approach, which takes into account these additional psychological and sociocultural factors, may be essential for enhancing outcomes in individuals with eating disorders.

## Discussion

### Overview of key findings

The findings of this systematic review highlight the complex interconnections of eating disorders, body image, emotion regulation, and self-esteem in adult populations. These elements do not operate in isolation; they interact in significant ways that can influence disordered eating. Notably, these interactions differed between clinical and nonclinical populations. Clinical populations consistently showed stronger associations between severe body dissatisfaction, emotional dysregulation, and lower self-esteem with clinical eating disorder diagnoses such as BED and BN. Nonclinical samples primarily showed associations between body dissatisfaction, subclinical disordered eating behaviors, and moderate emotional distress, suggesting differences in severity and mechanisms between clinical and nonclinical groups The six included studies demonstrate that factors such as feelings of dissatisfaction with one’s body, low self-esteem, and difficulty regulating emotions are significant contributors to disordered eating behaviors in adults. This connection is particularly important because it suggests that underlying psychological factors are crucial in understanding how eating disorders develop and persist. Such evidence supports recent research that focuses on the multifaceted nature of eating disorders, where psychological factors like body image and self-worth play critical roles in their development and continuation [25, 55]. This paper, therefore, underlines the importance of gaining a deeper understanding of the relationships and dynamics among the aforementioned factors, especially in adult populations that have been relatively overlooked in this area. This interrelation is further supported by evidence, noting that adults with eating disorders often display maladaptive perfectionism, highlighting a tendency to equate self-worth with body shape and weight, which increases vulnerability to these disorders. The role of negative affect, including feelings of sadness and anxiety, in exacerbating body dissatisfaction and poor self-esteem has been widely recognized as a risk factor for the onset of disordered eating behaviors. These findings point to the multifaceted etiology of eating disorders, highlighting the need for a more holistic approach to understanding and treating these complex conditions [11, 17, 69].

### The role of body image dissatisfaction

One important finding to be discussed is the impact of body image dissatisfaction on the development and severity of eating disorders. Individuals with BED and BN consistently displayed elevated levels of weight and shape concerns, which were strongly associated with their eating pathology. This aligns with theoretical perspectives on eating disorders, suggesting that body dissatisfaction can trigger unhealthy behaviors such as binge eating and compensatory actions [37, 71]. Interestingly, evidence also suggests that body image dissatisfaction is influenced by factors such as self-esteem and depression, particularly in women [7]. This implies that interventions that solely target body dissatisfaction may not be enough; instead, a more comprehensive approach that considers the psychological dimensions is necessary [51]. Body dissatisfaction may act as both a precursor and a consequence of eating disorders, suggesting a cyclical relationship that continues to maintain and exacerbate disordered eating. For this reason, enhancing body image flexibility—or the ability to accept and manage negative body-related thoughts—has been proposed as a protective factor against the development of eating disorders [9, 58, 72]. This perspective underscores the necessity of integrating therapeutic interventions that address not only body dissatisfaction but also enhance cognitive flexibility and emotional resilience to disrupt the cycle of disordered eating and body image issues effectively.

### Emotional dysregulation and eating disorders

The studies included in this review place significant emphasis on the role of emotion regulation, specifically the dysregulation of emotions and the link between emotional distress, such as depression, and disordered eating behaviors, especially in women. It is observed that some women may turn to dieting or binge eating as strategies to cope with their emotions [7]. There has been a clear link between negative emotional states, such as anxiety and depression, and an increase in binge eating episodes [36, 41]. Moreover, the study emphasized the importance of self-compassion in reducing the negative emotional impacts of higher BMI and body image dissatisfaction. By cultivating a compassionate attitude toward oneself, individuals may be able to decrease the risk of developing eating disorders [32]. The positive impact of self-compassion aligns with previous research showing its capacity to reduce the severity of eating disorder symptoms and enhance emotional well-being [6, 32]. Emotional dysregulation, especially when involving maladaptive strategies like emotional suppression and avoidance, has been identified as a key factor in both the development and persistence of eating disorders. In this context, mindfulness-based interventions have emerged as effective methods for helping individuals develop healthier emotion regulation, fostering awareness and nonjudgmental acceptance of emotions, and reducing disordered eating behaviors [31, 40, 66].

### Self-esteem and self-compassion

Turning to the role of self-esteem, it is evident that self-esteem plays a crucial part in the development of eating disorders, as research has consistently demonstrated. The findings indicate that interventions focused on enhancing self-esteem may be highly beneficial in preventing and treating eating disorders, especially in women who often use emotion-focused coping strategies [71]. The association between low self-esteem and disordered eating has been extensively documented, with studies showing that individuals with low self-esteem are more likely to engage in behaviors such as binge eating and purging to deal with feelings of inadequacy [29, 73]. However, the review also highlights the potential benefits of self-compassion compared to self-esteem in protecting against the negative psychological effects related to body image and eating disorders. This suggests a possible shift in therapeutic focus [32]. Further research suggests that self-compassion may provide greater resilience against the internalization of negative body image and eating disorder symptoms, indicating its significance alongside self-esteem [44]. Building self-esteem has been associated with long-term improvements in mental health and reductions in disordered eating behaviors. In terms of intervention strategies, acceptance and commitment therapy (ACT) has shown efficacy in improving self-esteem and reducing body dissatisfaction. On the other hand, self-compassion-based interventions may contribute to increased self-esteem and reductions in maladaptive perfectionism and body dissatisfaction [18, 19]. These therapeutic approaches illustrate the interplay between self-compassion and self-esteem, demonstrating how fostering self-compassion can mitigate risk factors associated with disordered eating behaviors, thereby providing a more resilient self-concept.

### Gender differences and sociocultural factors

Gender differences are a crucial factor in this review, as findings indicate that women tend to be more impacted by body image dissatisfaction and emotional distress, which can lead to more severe eating disorder behaviors. The findings suggest that interventions for specific genders are essential in addressing the distinct psychological challenges that women face concerning body image and eating disorders [5, 7]. This observation aligns with existing research that emphasizes how societal pressures related to body image can differ by gender. Women, in particular, are subjected to more intense scrutiny, leading to higher levels of body dissatisfaction and a greater risk of developing eating disorders [45, 65]. Women also often employ emotion-focused coping mechanisms, which can exacerbate these conditions. Consequently, the importance of gender-sensitive therapeutic approaches becomes clear [22]. While women are more frequently diagnosed with eating disorders, emerging research indicates that men are also affected by body dissatisfaction and disordered eating, although often in different forms, such as a focus on muscularity. Gendered social pressures, including the idealization of slimness for women and muscularity for men, can intensify body dissatisfaction and drive unhealthy eating and exercise habits. Therefore, developing a gender-sensitive approach is crucial for recognizing and effectively addressing the diverse ways body dissatisfaction manifests [12, 25, 47]. This review highlights the importance of considering the interconnected psychological factors of body image, self-esteem, and emotion regulation when treating eating disorders in adults. Future studies should prioritize the exploration of these variables in culturally diverse contexts and employ various research methodologies. This broader approach will enhance understanding and enable more effective treatment of eating disorders in adult populations. Additionally, incorporating techniques to strengthen self-compassion, emotion regulation, and body image perception into therapies and clinical practices may improve outcomes for individuals dealing with eating disorders [8]. Overall, the findings suggest that a holistic approach to treatment is crucial for effectively managing eating disorders in adults. Such an approach should include interventions that focus on enhancing self-esteem, regulating emotions, and improving body image perception.

### Limitations and future directions

This systematic review provides insights into the complex interplay between body image, self-esteem, emotion regulation, and eating disorders in adult populations. However, it is important to acknowledge several limitations. Firstly, all the included studies predominantly featured Western samples, which raises questions about the generalizability of the findings to non-Western cultures. The existing research may not fully capture how body image, self-esteem, and emotion regulation manifest in diverse cultural settings, especially in collectivistic societies where the understanding of body ideals and emotion regulation may differ significantly from Western norms. Consequently, future studies should aim to explore these variables in more culturally diverse populations, considering cross-cultural differences and including non-Western perspectives to provide a more comprehensive understanding of eating disorders in adulthood. Additionally, most of the studies included in this review had a predominance of female participants, which limits the conclusions that can be drawn about gender differences. Although the results indicate a significant impact of body image dissatisfaction and emotional dysregulation on women, more research is needed to examine how these factors affect men, especially considering the increasing prevalence of disordered eating and body dissatisfaction among males. A further limitation is that very few of the included studies examined anorexia nervosa, so we cannot conclude specifically about this disorder. Future studies should adopt a balanced gender representation and investigate the distinct experiences and challenges faced by both men and women with eating disorders, as well as those who identify outside the gender binary.

## Conclusion

This systematic review highlights the interdependence between eating disorders, body image, emotions, and self-esteem in adults. It emphasizes the interconnection of these variables and their substantial influence on the development and maintenance of disordered eating behaviors. Body image dissatisfaction and poor self-esteem may have a substantial influence, resulting in the adoption of unhealthy eating patterns. Emotional dysregulation exacerbates these issues, leading people to rely on maladaptive coping strategies such as excessive eating or severe dietary restrictions. The results suggest that successful treatment options should entail more than just addressing eating behaviors. An all-encompassing strategy is required, which simultaneously tackles self-esteem, emotional well-being, and body image perception. Integrating self-compassion into treatment may provide further benefits by helping clients mitigate the negative psychological consequences associated with eating disorders. The study emphasizes the need for tailoring therapies to address gender-specific difficulties since women seem to be more susceptible to the adverse effects of body image worries and mental discomfort. The gender disparity underscores the need for customizing therapy methods to account for the unique encounters of males and females.
